# Supplementary material for: Modeling glioblastoma heterogeneity as a dynamic network of cell states
Source: Mol Syst Biol. 2021 Sep 16;17(9):e10105. doi: 10.15252/msb.202010105 (PMC8444284; doi:10.15252/msb.202010105)
Supplement: Supplementary file 5 — Source Data for Figure 3 [file MSB-17-e10105-s001.zip › Figure3A_sourcedata/GSEA_3065/nosigdb_state2.GseaPreranked.1622718419215/OLSEN_SCPLIKE.html]

Details for gene set OLSEN\_SCPLIKE[GSEA]

|  || Dataset | state2 |
| Phenotype | NoPhenotypeAvailable |
| Upregulated in class | na\_neg |
| GeneSet | OLSEN\_SCPLIKE |
| Enrichment Score (ES) | -0.79156655 |
| Normalized Enrichment Score (NES) | -2.9395256 |
| Nominal p-value | 0.0 |
| FDR q-value | 0.0 |
| FWER p-Value | 0.0 |
Table: GSEA Results Summary

  

Fig 1: Enrichment plot: OLSEN\_SCPLIKE      
 Profile of the Running ES Score & Positions of GeneSet Members on the Rank Ordered List

  

| PROBE | GENE SYMBOL | GENE\_TITLE | RANK IN GENE LIST | RANK METRIC SCORE | RUNNING ES | CORE ENRICHMENT || 1 | CTNNAL1 |  |  | 532 | 0.176 | -0.0447 | No |
| 2 | S100B |  |  | 688 | 0.151 | -0.0508 | No |
| 3 | MT2A |  |  | 731 | 0.146 | -0.0451 | No |
| 4 | TOMM7 |  |  | 1996 | 0.065 | -0.1757 | No |
| 5 | RHOC |  |  | 2261 | 0.055 | -0.2001 | No |
| 6 | ZFP36L1 |  |  | 2320 | 0.053 | -0.2026 | No |
| 7 | CNN3 |  |  | 2349 | 0.052 | -0.2020 | No |
| 8 | S100A10 |  |  | 2608 | 0.044 | -0.2265 | No |
| 9 | ANXA5 |  |  | 2812 | 0.039 | -0.2455 | No |
| 10 | IFI16 |  |  | 3054 | 0.033 | -0.2690 | No |
| 11 | GAS7 |  |  | 3074 | 0.032 | -0.2688 | No |
| 12 | LIMA1 |  |  | 3279 | 0.028 | -0.2886 | No |
| 13 | CLIC4 |  |  | 4778 | 0.003 | -0.4484 | No |
| 14 | S100A11 |  |  | 5569 | -0.009 | -0.5322 | No |
| 15 | IFITM2 |  |  | 6128 | -0.019 | -0.5905 | No |
| 16 | CAVIN3 |  |  | 6155 | -0.019 | -0.5919 | No |
| 17 | SEMA3B |  |  | 6759 | -0.031 | -0.6542 | No |
| 18 | VAMP5 |  |  | 6835 | -0.032 | -0.6600 | No |
| 19 | MEF2C |  |  | 6861 | -0.033 | -0.6604 | No |
| 20 | ANXA1 |  |  | 7265 | -0.044 | -0.7004 | No |
| 21 | TIMP3 |  |  | 7309 | -0.045 | -0.7019 | No |
| 22 | QKI |  |  | 7478 | -0.050 | -0.7164 | No |
| 23 | ARPC1B |  |  | 7700 | -0.056 | -0.7361 | No |
| 24 | RASSF4 |  |  | 7851 | -0.061 | -0.7479 | No |
| 25 | S100A16 |  |  | 8127 | -0.073 | -0.7722 | No |
| 26 | BTG1 |  |  | 8156 | -0.075 | -0.7700 | No |
| 27 | PMP2 |  |  | 8160 | -0.075 | -0.7652 | No |
| 28 | ERBB3 |  |  | 8230 | -0.079 | -0.7671 | No |
| 29 | IFI6 |  |  | 8257 | -0.080 | -0.7643 | No |
| 30 | AHR |  |  | 8513 | -0.097 | -0.7849 | Yes |
| 31 | CRYAB |  |  | 8519 | -0.098 | -0.7786 | Yes |
| 32 | OLFML2A |  |  | 8569 | -0.101 | -0.7769 | Yes |
| 33 | AP1S2 |  |  | 8570 | -0.101 | -0.7699 | Yes |
| 34 | LGALS1 |  |  | 8680 | -0.110 | -0.7739 | Yes |
| 35 | VIM |  |  | 8691 | -0.110 | -0.7674 | Yes |
| 36 | CST3 |  |  | 8712 | -0.112 | -0.7618 | Yes |
| 37 | IFITM3 |  |  | 8720 | -0.113 | -0.7547 | Yes |
| 38 | ZEB2 |  |  | 8786 | -0.120 | -0.7534 | Yes |
| 39 | NTM |  |  | 8787 | -0.120 | -0.7451 | Yes |
| 40 | ITGA6 |  |  | 8820 | -0.125 | -0.7398 | Yes |
| 41 | SELENOM |  |  | 8855 | -0.129 | -0.7345 | Yes |
| 42 | ANXA2 |  |  | 8891 | -0.134 | -0.7290 | Yes |
| 43 | COL18A1 |  |  | 8909 | -0.137 | -0.7214 | Yes |
| 44 | PLP1 |  |  | 8931 | -0.139 | -0.7140 | Yes |
| 45 | PRNP |  |  | 9068 | -0.161 | -0.7175 | Yes |
| 46 | COL4A2 |  |  | 9069 | -0.161 | -0.7064 | Yes |
| 47 | S100A6 |  |  | 9097 | -0.166 | -0.6978 | Yes |
| 48 | SPTBN1 |  |  | 9111 | -0.169 | -0.6875 | Yes |
| 49 | PLAT |  |  | 9145 | -0.176 | -0.6789 | Yes |
| 50 | CD99 |  |  | 9224 | -0.203 | -0.6732 | Yes |
| 51 | GSN |  |  | 9238 | -0.206 | -0.6604 | Yes |
| 52 | COL4A1 |  |  | 9284 | -0.226 | -0.6496 | Yes |
| 53 | PNRC1 |  |  | 9294 | -0.229 | -0.6348 | Yes |
| 54 | IGFBP7 |  |  | 9295 | -0.229 | -0.6189 | Yes |
| 55 | LGALS3 |  |  | 9308 | -0.236 | -0.6039 | Yes |
| 56 | PMEPA1 |  |  | 9313 | -0.241 | -0.5877 | Yes |
| 57 | CTSD |  |  | 9320 | -0.246 | -0.5713 | Yes |
| 58 | CD9 |  |  | 9324 | -0.248 | -0.5545 | Yes |
| 59 | PMP22 |  |  | 9347 | -0.271 | -0.5381 | Yes |
| 60 | PTPRZ1 |  |  | 9351 | -0.272 | -0.5196 | Yes |
| 61 | CD59 |  |  | 9358 | -0.283 | -0.5007 | Yes |
| 62 | ITGB8 |  |  | 9362 | -0.294 | -0.4807 | Yes |
| 63 | PSAP |  |  | 9363 | -0.294 | -0.4604 | Yes |
| 64 | FN1 |  |  | 9364 | -0.294 | -0.4401 | Yes |
| 65 | EDNRB |  |  | 9369 | -0.301 | -0.4197 | Yes |
| 66 | B2M |  |  | 9383 | -0.331 | -0.3983 | Yes |
| 67 | SPARC |  |  | 9384 | -0.332 | -0.3754 | Yes |
| 68 | TIMP1 |  |  | 9387 | -0.334 | -0.3525 | Yes |
| 69 | ITGB1 |  |  | 9388 | -0.339 | -0.3290 | Yes |
| 70 | GPM6B |  |  | 9389 | -0.344 | -0.3053 | Yes |
| 71 | CD63 |  |  | 9394 | -0.353 | -0.2813 | Yes |
| 72 | ITM2B |  |  | 9399 | -0.371 | -0.2561 | Yes |
| 73 | APP |  |  | 9412 | -0.426 | -0.2280 | Yes |
| 74 | VMP1 |  |  | 9417 | -0.448 | -0.1974 | Yes |
| 75 | DST |  |  | 9422 | -0.485 | -0.1644 | Yes |
| 76 | MALAT1 |  |  | 9436 | -0.735 | -0.1150 | Yes |
| 77 | IGFBP5 |  |  | 9438 | -0.797 | -0.0601 | Yes |
| 78 | NEAT1 |  |  | 9439 | -0.871 | 0.0001 | Yes |
Table: GSEA details [plain text format]

  

Fig 2: OLSEN\_SCPLIKE: Random ES distribution      
 Gene set null distribution of ES for **OLSEN\_SCPLIKE**
